# Trace Amine-Associated Receptor 1 Modulates the Locomotor and Sensitization Effects of Nicotine

**DOI:** 10.3389/fphar.2018.00329

**Published:** 2018-04-06

**Authors:** Ilya Sukhanov, Mariia Dorofeikova, Antonina Dolgorukova, Artem Dorotenko, Raul R. Gainetdinov

**Affiliations:** ^1^Laboratory of Behavioral Pharmacology, Valdman Institute of Pharmacology, Pavlov First Saint Petersburg State Medical University, Saint Petersburg, Russia; ^2^Laboratory of Neurochemical Pharmacology, Neuroscience and Brain Technologies, Fondazione Istituto Italiano di Technologia, Genoa, Italy; ^3^Institute of Translational Biomedicine, Saint Petersburg State University, Saint Petersburg, Russia; ^4^Skolkovo Institute of Science and Technology, Skolkovo Innovation Center, Moscow, Russia

**Keywords:** TAAR1, nicotine, dopamine, drug addiction, sensitization, locomotor activity

## Abstract

Trace amine-associated receptor 1 (TAAR1) has emerged as a promising target for addiction treatments because it affects dopamine transmission in the mesolimbic pathway. TAAR1 is involved in the effects of addictive drugs, such as amphetamines, cocaine and ethanol, but the impact of TAAR1 on the effects of nicotine, the psychoactive drug responsible for the development and maintenance of tobacco smoking, has not yet been studied. This study was performed to investigate the possible modulatory action of TAAR1 on the effects of nicotine on locomotor behaviors in rats and mice. Pretreatment with the TAAR1 agonist RO5263397 dose-dependently decreased nicotine-induced hyperlocomotion in rats habituated to locomotor boxes, prevented the development of nicotine sensitization and blocked hypermotility in nicotine-sensitized rats at the highest tested dose (10 mg/kg). The lack of TAAR1 failed to affect the effects of nicotine on the locomotion of mutant mice. Based on the results of the present study, TAAR1 activation attenuates the locomotion-stimulating effects of nicotine on rats. These results further support the previously proposed hypothesis that TAAR1 is a promising target for the prevention and treatment of drug addiction. Further studies aimed at analyzing the effects of TAAR1 agonists on animal models of nicotine addiction are warranted.

## Introduction

Tobacco smoking is the most common type of drug dependence worldwide (Anthony et al., 1994). The nicotine (NIC) contained in tobacco is the main reason for the development and maintenance of tobacco smoking (Stolerman and Jarvis, 1995), which is one of the principal risk factors for leading causes of human mortality, such as cancer, chronic obstructive pulmonary disease, and coronary heart disease (Murray and Lopez, 1997). In 2015, the World Health Organization estimated that more than 1.1 billion individuals smoked worldwide (World Health Organization, 2015). Estimates predict that approximately 1.5 to 1.9 billion individuals will be smokers in 2025. Current pharmacological approaches for tobacco smoking therapy are not sufficiently effective and mainly include substitution therapy. The current lack of methods to control NIC abuse has prompted researchers to search for new approaches.

According to the literature, the mesocorticolimbic dopaminergic pathway may play essential roles in hedonic activation, associative learning and incentive salience to stimuli associated with the effects of NIC and other addictive drugs (Robinson and Berridge, 2000; Markou, 2008). Trace amine-associated receptor 1 (TAAR1) is expressed in the ventral tegmental area (Bunzow et al., 2001) and modulates dopaminergic activity mainly through D2 receptor mechanisms (Espinoza et al., 2011; Leo et al., 2014; Sukhanov et al., 2014; Asif-Malik et al., 2017). Hyperactivity induced by psychostimulants is considered to reflect, to a large extent, the behavioral manifestation of increased dopamine activity in the mesolimbic dopaminergic pathway (Zetterstrom et al., 1983). TAAR1 knockout (TAAR1-KO) mice are more sensitive to hyperactivity induced by dopaminergic psychostimulants, such as amphetamine (Wolinsky et al., 2007; Sotnikova et al., 2008; Achat-Mendes et al., 2012), methamphetamine (Achat-Mendes et al., 2012), or 3,4-Methylenedioxymethamphetamine (Sotnikova et al., 2008; Di Cara et al., 2011) than wild type (WT) control animals. Analogously, TAAR1 agonists attenuate hyperlocomotion induced by both dopamine psychostimulants (such as cocaine and amphetamine) and other addictive drugs that also show psychostimulant activity (Danysz et al., 1994; Sukhanov et al., 2004), such as phencyclidine, in mice (Revel et al., 2011, 2012a, 2013), Wistar rats (Revel et al., 2012b), and TAAR1-overexpressing mice (Revel et al., 2012b). Consistent with these findings, the TAAR1 partial agonists RO5263397 and RO5203648 attenuate cocaine and methamphetamine sensitization (Jing et al., 2014; Thorn et al., 2014a,b). Furthermore, RO5263397 (Thorn et al., 2014a) and RO5166017 (Liu et al., 2017) attenuate the development of cocaine conditioned place preference (CPP).

Lynch et al. (2013), TAAR1-KO mice were more sensitive to the sedative-like effects of ethanol and drank more ethanol, but not saccharine, in a two-bottle test than WT control animals. Importantly, the lack of TAAR1 did not alter ethanol clearance in mice (Lynch et al., 2013). Similar results with voluntary methamphetamine drinking were also obtained in TAAR1-KO mice: the mutant animals consumed more of the methamphetamine solution than controls (Harkness et al., 2015). Additionally, the lack of TAAR1 increased the animals’ sensitivity to contextual stimuli associated with the addictive drug in the prime-induced reinstatement of amphetamine CPP, context-dependent amphetamine sensitization and methamphetamine CPP under suboptimal conditions (Achat-Mendes et al., 2012; Sukhanov et al., 2016).

Accumulating evidence from self-administration studies supports a possible regulatory role for TAAR1 in the behavioral properties of addictive drugs. RO5203648 and RO5256390 decrease cocaine self-administration in Long Evans rats (Revel et al., 2012b; Pei et al., 2015). Moreover, RO5263397 and RO5203648 reduce the number of methamphetamine injections in the self-administration paradigm in rats (Jing et al., 2014; Cotter et al., 2015). In control experiments, TAAR1 agonists do not affect operant schedules reinforced with food or saccharine (Pei et al., 2014; Cotter et al., 2015; Ferragud et al., 2017). Interestingly, in a few substitution tests, RO5203648 was not able to maintain the intravenous self-administration that had previously been reinforced by cocaine and methamphetamine (Cotter et al., 2015; Pei et al., 2017). Additionally, TAAR1 agonists attenuate the reinstatement of cocaine and methamphetamine, but not saccharine, self-administration (Jing et al., 2014; Pei et al., 2014, 2017), although the combination of RO5263397 and extinction does not affect the relapse of cocaine self-administration in rats (Liu et al., 2017).

Although TAAR1 has been shown to modulate the effects of addictive drugs, the impact of TAAR1 on the effects of NIC has not yet been studied. This study was conducted to investigate the impact of TAAR1 on the effects of NIC on locomotion in animals. The main questions were: (a) Does the partial TAAR1 agonist RO5263397, which is used in most addiction-related studies, decrease hyperlocomotion in rats produced by acute NIC administration? (b) Does RO5263397 prevent the development of NIC locomotor sensitization? (c) Does RO5263397 block NIC hyperactivity in NIC-sensitized rats? (d) How does the lack of TAAR1 modulate the effects of NIC on mice?

Preliminary results of this study were presented at the 30th European College of Neuropsychopharmacology Congress (September 2–5, 2017, Paris, France) and were published as a conference abstract (Sukhanov et al., 2017).

## Materials and Methods

### Animals

Drug and test naïve Wistar rats (3–4 months old at the beginning of experiments) were purchased from the State Breeding Farm “Rappolovo” (Saint Petersburg, Russia). WT and TAAR1-KO mice were derived from crossing (over 10 generations) heterozygous TAAR1 C57BL6/129SvJ animals (Wolinsky et al., 2007). Heterozygous mice were not analyzed in this study. All rodents were housed under standard laboratory conditions on a 12 h light/dark cycle (lights on at 08:00 h) in a room with a temperature of 21 ± 2°C and 50 ± 20% humidity. Rats were housed in groups of four-five in standard T IV cages. Mice were housed in groups of 3–5 per standard T III cage. During the experiments, rats and mice had free access to drinking water and food. All experiments were performed during the light period of the light/dark cycle after at least 1 week of habituation to the animal facility. Cages and water bottles were replaced weekly.

Experimental protocols were approved by the local Animal Care and Use committee (First Pavlov State Saint Petersburg Medical University, # 100_
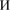
Φ1_012017/3_900, tests in rats) or the Italian Ministry of Health (permit #17 BIS/2014, experiment in TAAR1-KO mice).

### Drugs

RO5263397, the partial TAAR1 agonist, was synthesized at F. Hoffmann-La Roche (Basel, Switzerland). RO5263397 was dissolved in 1% Tween 80. (-)-Nicotine hydrogen tartrate salt (Sigma-Aldrich, Co., St. Louis, MO, United States) was dissolved in sterile saline.

Fresh solutions of the drugs were prepared daily and administered to rats in a dosing volume of 1 ml/kg and to mice in a volume of 10 ml/kg.

### Impacts of RO5263397 on the Locomotor Effects of Acute NIC Administration in Rats

Locomotor behaviors of rats (*n* = 8) were evaluated in two sets of five identical boxes (25 cm × 35.5 cm × 34 cm), each with transparent Plexiglas walls and a non-transparent plastic floor enclosed within sound-attenuating ventilated cubicles. The light intensity inside the apparatus was 30–40 lx. Each box was equipped with 11 pairs of photocell-based infrared sensors. Three pairs of photocell units located 5 cm above the bottom of the box were used to record horizontal activity. In addition, eight pairs of photocell units were placed 14 cm above the floor to record vertical activity. The total number of repetitive vertical beam breaks (vertical activity) and sequential beam breaks (ambulations) was recorded by MED-PC software (MED Associates, East Fairfield, VT, United States).

A group of eight rats was tested in the apparatus after a habituation period (baseline period). During this period, animals were placed in locomotor activity boxes for 60 min per day, 6 days a week to habituate to the environment. After all rats exhibited a stable locomotor activity level (i.e., the assessed parameters did not change by more than 10% within three consecutive days), drug tests were initiated. The order in which the tested doses were administered was based on a within-subject Latin Square design. Drug tests were separated by at least 72 h. Rats were placed in activity chambers for 60 min immediately after the injections. NIC was administered subcutaneously (s.c.) at a dose of 0.4 mg/kg (base). RO5263397 (1, 3, or 10 mg/kg) or vehicle were administered intraperitoneally (i.p.).

### NIC Sensitization in Rats

**FIGURE 1:**
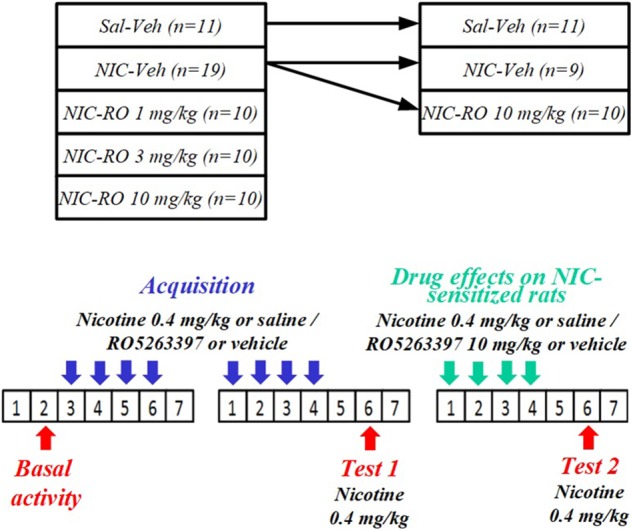
The schema of NIC sensitization in rats.

Locomotor behaviors of rats (*n* = 60) were evaluated in the apparatus described above. Before the acquisition of NIC sensitization, rats were divided into five groups according to baseline locomotor activity: vehicle-saline (*n* = 11), vehicle-NIC (*n* = 19), 1 mg/kg RO5263397-NIC (*n* = 10), 3 mg/kg RO5263397-NIC (*n* = 10), and 10 mg/kg RO5263397-NIC (*n* = 10). As shown in **Figure 1**, the acquisition of NIC sensitization included eight sessions in the activity chambers. Prior to every acquisition session, rats were i.p. pretreated with either RO5263397 or vehicle and then injected with either NIC 0.4 mg/kg (base) or saline s.c. 48 h after the final acquisition session. Animals were then tested for NIC sensitization (Test 1). All animals were administered NIC injections immediately before being exposed to the activity cages for 60 min. The vehicle-NIC group was divided into two subgroups to evaluate the effects of RO5263397 on NIC-sensitized rats. Before each of the four additional sessions, the animals were injected with either RO5263397 (10 mg/kg) (*n* = 10), vehicle (*n* = 9), or NIC (0.4 mg/kg, base). Forty-eight hours after the fourth session, rats were again tested for NIC sensitization (Test 2) using the same method as in Test 1.

### Locomotor Effects of NIC on TAAR1-KO Mice

The effects of NIC on the locomotor behaviors of independent groups of TAAR1 KO and WT mice (0 mg/kg: *n* = 7 and *n* = 8; 0.3 mg/kg: *n* = 8 and *n* = 8; 0.4 mg/kg: *n* = 7 and *n* = 8; 0.6 mg/kg: *n* = 7 and *n* = 8; 0.8 mg/kg: *n* = 8 and *n* = 8; 1 mg/kg: *n* = 6 and *n* = 9; 1.5 mg/kg: *n* = 8 and *n* = 8; for WT and TAAR1-KO mice, respectively) were examined using an automated Omnitech Digiscan apparatus (AccuScan Instruments, Columbus, OH, United States) under illuminated conditions. The apparatus included four open field monitors. Each open field monitor consisted of sets of 16 light beams arrayed in the horizontal plane on the X and Y axes. The hardware detected the number of times each animal broke the beams, which allowed the software to determine the location of the mouse in the cage. Cages were divided into four compartments (20 cm × 20 cm). The animals were tested individually for 90 min with 5-min intervals. Horizontal and vertical locomotion were measured by determining the number of beam breaks. Before NIC administration, mice were habituated to the activity monitor for 30 min. After the s.c. injection of NIC (0.3–1.5 mg/kg, base), the locomotor activity of the animals was recorded for an additional 60 min.

### Statistical Analyses

We compared the number of ambulations and vertical activity measured on the last day before the start of the pharmacological tests and after NIC and vehicle administration using the Wilcoxon signed-rank test to measure the effects of NIC on locomotor activity in rats. The results of the analysis of the effects of RO5263397 (independent variable) on locomotor activity (dependent variables) in rats that received an acute NIC treatment were analyzed using one-way Friedman’s repeated measures (RM) analysis of variance (ANOVA) of ranks followed by Dunnett’s *post hoc* test.

For the acquisition of NIC sensitization, the baseline locomotor activity (dependent variables) of different rat groups (independent variable) was subjected to one-way Kruskal–Wallis ANOVA of ranks. Following the rank transformation, baseline locomotor activity and locomotor activity during Test 1 were analyzed using two-way RM ANOVA followed by Bonferroni’s *post hoc* test. The data from Test 1 are presented as % of baseline locomotor activity and were subjected to one-way Kruskal–Wallis ANOVA of ranks followed by Dunn’s *post hoc* test to exclude a possible effect of baseline locomotor activity.

Baseline locomotor activity (independent variables) of the rat subgroups (dependent variable) and locomotor activity during Test 1 were analyzed using one-way Kruskal–Wallis ANOVA of ranks followed by Dunn’s *post hoc* test to evaluate the effect of RO5263397 on NIC-sensitized rats. Following rank transformation, the numbers of ambulations and vertical activity during four sessions were subjected to two-way RM ANOVA followed by series of one-way Kruskal–Wallis ANOVAs. Locomotor data measured at the baseline and in Tests 1 and 2 were also rank transformed and analyzed using two-way RM ANOVA followed by Bonferroni’s *post hoc* test.

Baseline locomotor mice activity (independent variables) was subjected to Mann–Whitney rank sum test (*U*-test). Following the rank transformation, the data were analyzed using two-way RM ANOVA followed by Bonferroni’s *post hoc* test to analyze the effects of NIC (dependent variables) on the mice. We compared locomotor activity TAAR1-KO and WT mice measured after NIC (1.5 mg/kg) using *U*-test to measure the effects of the highest tested NIC on the horizontal and vertical activity in the mice.

The alpha value was set to 0.05. All statistical analyses were performed using SigmaPlot 12.5 (Systat Software, Inc., San Jose, CA, United States) or IBM SPSS Statistics 21 software (IBM, Armonk, NY, United States).

## Results

### RO5263397 Dose-Dependently Attenuates NIC-Induced Hyperactivity in Rats

**FIGURE 2:**
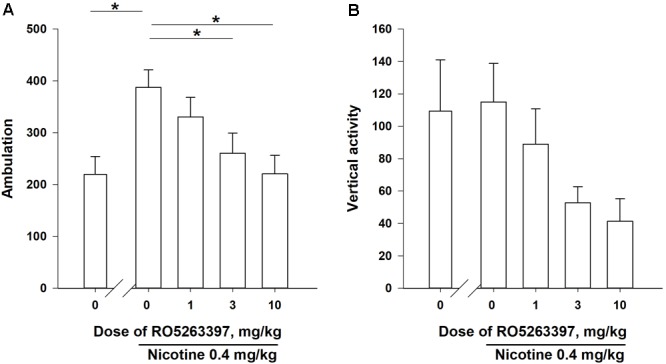
The effects of RO5263397 (1.0–10.0 mg/kg, i.p.) on acute NIC-induced locomotor activity in rats (**A** is number of ambulations, **B** is vertical activity). The order of the administration of the test doses in the experiments was based on a within-subject Latin Square design and the drug tests were separated by at least 72 h intervals. All injections were performed immediately before the experimental sessions. Data are presented as means ± SEM. *n* = 8 animals per data point. ^∗^*P* < 0.05 compared with the vehicle control treatment, as analyzed using Dunnett’s *post hoc* test.

As presented in **Figure 2**, NIC (0.4 mg/kg, base) administration increased the number of ambulations (*P* < 0.05), but did not affect vertical activity (*P* = 0.18). Thus, only the number of ambulations was used to evaluate the effects of RO5263397 on NIC-induced locomotor activity. According to the one-way Friedman RM ANOVA, RO5263397 dose-dependently attenuated NIC-induced hyperactivity (χ^2^ = 12.300, df = 3, *P* < 0.01). Dunnett’s *post hoc* test revealed that the effects of RO5263397 were statistically significant at the doses of 3 and 10 mg/kg (*P* < 0.05).

### Pretreatment With RO5263397 Prevents NIC Sensitization

Prior to sensitization, we evaluated the rats’ baseline locomotor activity. According to the one-way Kruskal–Wallis ANOVAs of ranks, differences were not observed between the groups of animals (number of ambulations: χ^2^= 3.667, df = 4, *P* = 0.45; vertical activity: χ^2^= 6.098, df = 4, *P* = 0.19).

**FIGURE 3:**
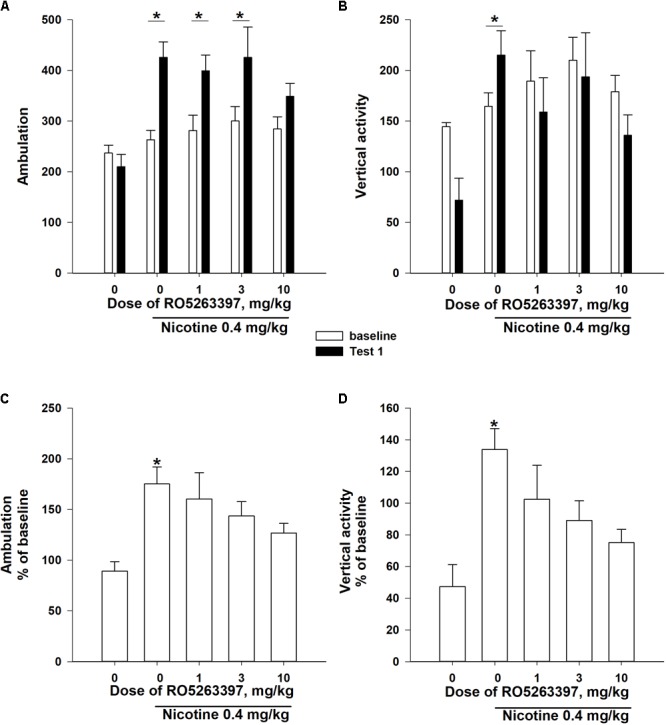
The effects of RO5263397 (1.0–10.0 mg/kg, i.p.) on the development of sensitization to the locomotor-stimulating effects of NIC on rats. Before the acquisition of NIC sensitization, rats were divided into five groups according to the baseline locomotor activity. The acquisition of NIC sensitization included eight sessions in the activity chambers. Prior to every acquisition session, rats were i.p. pretreated with either RO5263397 or vehicle and then injected with either NIC 0.4 mg/kg (base) or saline s.c. During Test 1, all animals were injected with NIC immediately before being exposed to the activity cages for 60 min. Data are presented as the means ± SEM. ^∗^*P* < 0.05 compared with the baseline activity of the indicated group **(A,B)** or the vehicle control treatment, as analyzed using Bonferroni’s **(A,B)** or Dunn’s **(C,D)** *post hoc* tests.

The two-way ANOVAs of the rank-transformed Test 1 data revealed significance effects of the between-subject factor “group” [number of ambulations: *F*(4,55) = 6,387, *P* < 0.001; vertical activity: *F*(4,55) = 2,871, *P* < 0.05], the within-subject factor “sensitization” [number of ambulations: *F*(1,55) = 28,469, *P* < 0.001; vertical activity: *F*(1,55) = 8,008, *P* < 0.01] and a significant interaction between these factors [number of ambulations: *F*(1,55) = 3,717, *P* < 0.05; vertical activity: *F*(1,55) = 4,354, *P* < 0.01]. As presented in **Figures 3A,B**, the administration of NIC (0.4 mg/kg, base) for eight sessions increased the number of ambulations and the vertical activity in the rats in the vehicle-NIC group (*P* < 0.05, Bonferroni’s *post hoc* test), but the development of NIC sensitization was prevented in the groups treated with 1 mg/kg (vertical activity: *P* = 0.17), 3 mg/kg (vertical activity: *P* = 0.08), and 10 mg/kg RO5263397 (number of ambulations: *P* = 0.06).

Additionally, the one-way Kruskal–Wallis ANOVAs of ranks revealed a significant effect of the between-subject factor “group” on the Test 1 locomotor activity, presented as % of baseline locomotor activity (**Figures 3C,D**: number of ambulations: χ^2^= 16,441, df = 4, *P* < 0.01; vertical activity: χ^2^= 16,651, df = 4, *P* < 0.01). Dunn’s *post hoc* test revealed that the pretreatment with all doses of RO5263397 blocked the development of NIC locomotor sensitization (**Figures 3C,D**; *P* > 0.05).

### RO5263397 Attenuates NIC-Induced Hyperactivity in NIC-Sensitized Rats

**FIGURE 4:**
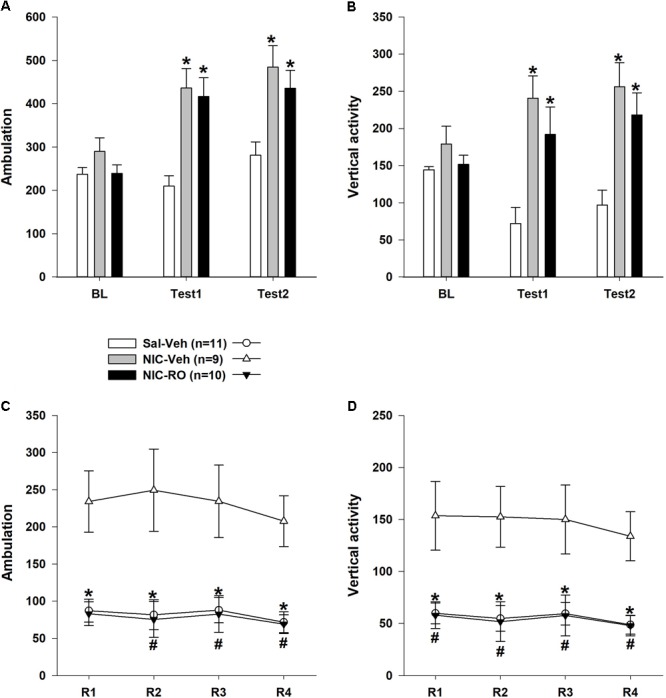
The effects of repeated RO5263397 (10 mg/kg) administrations on the locomotor activity of rats sensitized to stimulatory effects of nicotine on locomotion. **(A,B)** Prior to each of the four additional sessions, animals were injected with either RO5263397 or vehicle and NIC. **(C,D)** Subsequently, rats were again tested for NIC sensitization (Test 2) using the methods described for Test 1. Data are presented as the means ± SEM. *n* = 9–11 animals per data point. ^∗^*P* < 0.05 compared with the vehicle control treatment subgroup, as analyzed using Dunn’s **(A,B)** Bonferroni’s **(C,D)** *post hoc* tests. ^#^*P* < 0.05 compared with the subgroup treated with nicotine and vehicle, as analyzed using Bonferroni’s *post hoc* tests.

As shown in **Figures 4A,B**, the baseline locomotor activity of the animals did not differ (one-way Kruskal–Wallis ANOVA on ranks: number of ambulations: χ^2^= 3,198, df = 2, *P* = 0.20; vertical activity: χ^2^= 0,730, df = 2, *P* = 0.69). Based on the results from Test 1, NIC induced distinct locomotor hyperactivity in both NIC-treated subgroups. The one-way Kruskal–Wallis ANOVA of ranks revealed a significant effect of the between-subject factor “group” (number of ambulations: χ^2^= 3,198, df = 2, *P* = 0.20; vertical activity: χ^2^= 0,730, df = 2, *P* = 0.69). Dunn’s *post hoc* test revealed that both NIC-treated subgroups were more active than rats in the vehicle-saline group (*P* < 0.05). We also evaluated the effects of the combination of RO5263397 and NIC on NIC sensitization retention because previous studies supported the hypothesis that pharmacological agents might reverse the sensitization to the locomotor effects of drugs (Li et al., 2000; Zhang et al., 2006, 2007; Carrera et al., 2011). As illustrated in **Figures 4A,B**, the pharmacological treatment did not affect the locomotor activity of the animals during Test 2. The two-way ANOVAs of the rank transformed data revealed significant effects of the between-subject factor “group” [number of ambulations: *F*(2,27) = 13,931, *P* < 0.001; vertical activity: *F*(2,27) = 11,120, *P* < 0.001], the within-subject factor “treatment” [number of ambulations: *F*(2,51) = 19,635, *P* < 0.001; vertical activity: *F*(2,54) = 2,008, *P* = 0.09] and a significant interaction between these factors [number of ambulations: *F*(2,51) = 4,224, *P* < 0.01; vertical activity: *F*(2,54) = 4,826, *P* < 0.01]. Similar to Test 1, the *post hoc* analyses revealed that both NIC-treated subgroups were more active than rats in the vehicle-saline group during Test 2 (*P* < 0.05), but differences between these subgroups were not significant (*P* = 1.0).

As shown in **Figures 4C,D**, the RO5263397 pretreatment blocked the effects of NIC on the NIC-sensitized animals’ locomotor activity. This effect of RO5263397 was observed during all reversal sessions. The two-way ANOVAs revealed a significant effect of the between-subject factor “group” [number of ambulations: *F*(2,27) = 18,494, *P* < 0.001; vertical activity: *F*(2,27) = 8,588, *P* < 0.01]. The effects of the within-subject factor “treatment” [number of ambulations: *F*(3,70) = 0,979, *P* = 0.39; vertical activity: *F*(3,81) = 1,751, *P* = 0.16] and the interaction between the factors “group” and “treatment” [number of ambulations: *F*(5,70) = 1,180, *P* = 0.33; vertical activity: *F*(6,81) = 0,367, *P* = 0.90] were not significant. For more in-depth analyses, a series of one-way Kruskal–Wallis ANOVAs of ranks was performed. All ANOVAs revealed a significant effect of the between-subject factor “group” on locomotor activity (*P* < 0.05). Dunn’s *post hoc* test revealed that RO5263397 administration blocked the stimulatory effect of NIC on both the number of ambulations and vertical activity in the NIC-sensitized rats (*P* < 0.05 compared to the locomotor activity of the NIC-vehicle subgroup on the corresponding day).

### The Lack of TAAR1 Does Not Affect the Effects of NIC on the Locomotor Activity of Mice

**FIGURE 5:**
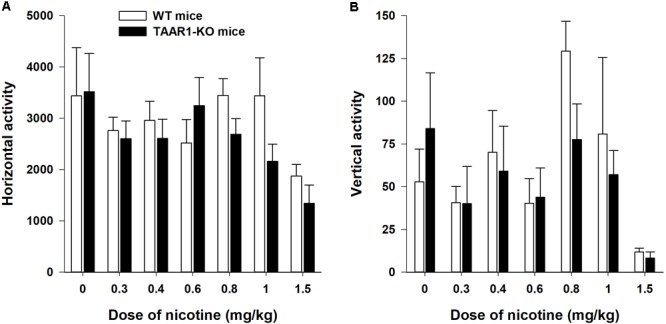
Effects of NIC on the locomotor activity (**A** is horizontal activity, **B** is vertical activity) of TAAR1-KO mice. After a 30 min habituation period, the mice were injected with NIC (0.3–1.5 mg/kg) and immediately placed in the open field apparatus for an additional 60 min. Data are presented as the means ± SEM.

The TAAR1-KO and control mice exhibited similar levels of horizontal and vertical activities after the administration of the vehicle (**Figure 5**). The *U*-test did not reveal any effects of the mutation on the locomotor activity of the vehicle-treated mice (horizontal activity: *P* = 0.88; vertical activity: *P* = 0.61). The two-way ANOVAs of the rank transformed data revealed a significant effect of the between-subject factor “dose” [horizontal activity: *F*(6,94) = 3,307, *P* < 0.01; vertical activity: *F*(6,94) = 6,089, *P* < 0.001]. The *post hoc* analyses (Dunnett’s test) indicated that only the highest tested dose of NIC (1.5 mg/kg) significantly decreased the locomotor activity (*P* < 0.01). The lack of TAAR1 did not alter the effects of NIC on the locomotor activity of the mice [the main effect of the factor “mutation”: *F*(1,94) = 1,222, *P* = 0.27; *F*(1,94) = 0,969, *P* = 0.77 for horizontal and vertical activities, respectively]. The *U*-test did not reveal any effects of the mutation on the hypolocomotor action of the highest tested dose of NIC (1.5 mg/kg) (horizontal activity: *P* = 0.16; vertical activity: *P* = 0.23).

## Discussion

In the present study, we investigated the impact of the partial TAAR1 agonist RO5263397 on the acute and chronic effects of NIC on locomotor activity. NIC is considered a psychostimulant that is known to enhance the performance of humans on motor, attentional, and memory tasks (Heishman et al., 2010). The data on the effects of NIC on rodent locomotor behaviors are somewhat controversial. Acute NIC administration reliably induces hyperactivity in rats (Clarke and Kumar, 1983; Green et al., 2003), but decreases locomotor activity in mice in some studies (Morrison, 1969; Bernardi and Spanagel, 2014). In the present study, the partial TAAR1 agonist RO5263397 reversed the hyperactivity induced by acute NIC administration in rats. These results are consistent with other studies of the effects of psychostimulants on rats. In fact, RO5203648 attenuates hyperlocomotion induced by the psychostimulants cocaine and methamphetamine (Revel et al., 2012b; Cotter et al., 2015). However, in another study, RO5263397 (3.2 and 10 mg/kg) did not affect locomotion induced by cocaine (15 mg/kg) administration (Thorn et al., 2014b). Interestingly, RO5263397 also blocked NIC hyperactivity in rats sensitized to the stimulatory actions of NIC and repeated RO5263397 administration did not affect this action in the present study.

Consistent with the incentive-sensitization theory of drug addiction, repeated exposure to addictive drugs induces the sensitization of mesocorticolimbic dopamine neurons (Robinson and Berridge, 2000). In this form of neuroadaptation, drug-related stimuli would become more effective at inducing dopamine efflux in the mesocorticolimbic areas and in triggering cravings (Di Chiara and Bassareo, 2007). Sensitization to the locomotor-stimulating actions of addictive drugs in animals has been proposed to mirror the sensitization of the mesocorticolimbic dopamine pathway (Vanderschuren and Kalivas, 2000; Vezina and Leyton, 2009). Similar to other addictive drugs, repeated NIC treatments induce robust locomotor sensitization in rats (Shoaib and Stolerman, 1992; Kempsill and Pratt, 2000). In our study, RO5263397 prevented the development of NIC sensitization. Similarly, in previous studies, RO5263397 and RO5203648 were able to prevent methamphetamine and cocaine sensitization (Jing et al., 2014; Thorn et al., 2014a,b). Studies in TAAR1-KO mice supported the regulatory role of TAAR1 in the acquisition of sensitization to the motor-stimulating actions of psychostimulants. Mutant mice are more sensitive to the sensitizing actions of amphetamine and methamphetamine than control mice (Achat-Mendes et al., 2012; Sukhanov et al., 2016).

The mechanism by which TAAR1 blocks NIC-induced locomotor hyperactivity has not yet been well elucidated. As described above, the NIC-induced hyperactivity observed in rats is considered to reflect, to a large extent, a behavioral manifestation of increased dopamine activity in the mesolimbic pathway (Kleijn et al., 2011). The hypermotility is mediated by the activation of post-synaptic D1-like and D2-like receptors (Jaber et al., 1996), whereas the activation of presynaptic D2-like receptors is involved in the negative feedback mechanisms regulating dopamine release, thereby decreasing locomotor activity (Strombom, 1976; Gainetdinov et al., 1996; De Mei et al., 2009). Based on the results from *in vitro* studies, TAAR1 might form functional heterodimers with D2-like dopamine receptors (Espinoza et al., 2011). In fact, haloperidol, a D2 antagonist, significantly reduces catalepsy in TAAR1-KO mice (Espinoza et al., 2011). Moreover, the stimulation of locomotor activity by quinpirole, a selective agonist of dopamine D2/3 receptors, was enhanced in TAAR1-KO mice (Espinoza et al., 2015). Recently, the full TAAR1 agonist RO5256390 was shown to completely prevent cocaine-induced dopamine efflux in the mesolimbic pathway (Asif-Malik et al., 2017) because of the interaction of the inhibitory presynaptic D2-like autoreceptor with TAAR1 (Leo et al., 2014).

Consistent with previous reports, NIC decreased horizontal and vertical locomotor activity in mice in the present study. According to our results, the lack of TAAR1 did not affect the NIC-mediated reduction in locomotor activity. The absence of changes in the effects of NIC on the locomotor activity of TAAR1-KO mice observed in our study might be due to the involvement of non-dopaminergic mechanisms. Non-dopaminergic mechanisms may be responsible for the acute locomotor hypomotility induced by NIC in mice (Damaj and Martin, 1993a,b) or simply reflect technical problems in detecting the small stimulatory action of nicotine in mice that have a higher level of basal locomotor activity than rats, as has been observed for D2R agonists (Espinoza et al., 2015). In contrast to NIC, psychostimulants such as amphetamines and cocaine induce clear locomotor hyperactivity in mice, and the lack of TAAR1 increases the sensitivity of mice to the locomotor-stimulating actions of these drugs (Wolinsky et al., 2007; Sotnikova et al., 2008; Di Cara et al., 2011; Achat-Mendes et al., 2012). Interestingly, treatment with ethanol, which, at certain doses, decreases motor activity in mice to a similar extent as NIC, induces more prominent hypolocomotion in TAAR1-KO mice than in control mice (Lynch et al., 2013).

In summary, based on the data from the present study, TAAR1 activation attenuates the locomotor-stimulating and sensitization effects of NIC on rats. After the submission of this report, an exciting paper was published that presented similar observations of the locomotor effects of NIC (Liu et al., 2018). Moreover, in this comprehensive and carefully executed study, a number of other behavioral and neurochemical parameters reflecting the effects of NIC were evaluated under conditions in which TAAR1 activity was modulated. In particular, the systemic administration of the TAAR1 agonists RO5166017 and RO5263397 attenuates nicotine self-administration, reinstates nicotine-seeking behaviors, and increases the elasticity of the nicotine demand curve. Importantly, TAAR1 activation reduces NIC-induced dopamine release in the nucleus accumbens (NAc) and intra-NAc infusions of a TAAR1 agonist are sufficient to block nicotine self-administration reinstatement in rats. Furthermore, TAAR1-knockout rats exhibit a higher propensity toward cue-induced and drug-induced reinstatement of nicotine-seeking behaviors. These observations convincingly revealed that the modulation of TAAR1 activity regulates nicotine-induced addictive-like behaviors (Liu et al., 2018). Taken together, these two independent reports not only further support the previously proposed hypothesis that TAAR1 is a promising target to modulate the behavioral effects of addictive drugs but also make a strong case for considering TAAR1 as a novel target for the treatment of nicotine addiction. Further studies aimed at analyzing the effects of TAAR1 agonists on animal models of NIC addiction are warranted.

## Author Contributions

IS and RG designed and directed the project. IS, MD, ADol, and ADor performed the experiments. IS and MD analyzed the data. All authors discussed the results and contributed to the final manuscript. IS wrote the manuscript.

## Conflict of Interest Statement

The authors declare that the research was conducted in the absence of any commercial or financial relationships that could be construed as a potential conflict of interest.
